# A NOVEL TIME USE APPROACH ON SUCCESSFUL AGING: RACIAL AND GENDER DISPARITIES IN DAILY PRODUCTIVE ENGAGEMENT

**DOI:** 10.1093/geroni/igae098.2201

**Published:** 2024-12-31

**Authors:** Jason Wong, Siyao Lu, Yifan Lou, Emma Zang, Deborah Carr

**Affiliations:** Yale University, New Haven, Connecticut, United States; Yale University, New Haven, Connecticut, United States; Virginia Commonwealth University, New Haven, Virginia, United States; Yale University, New Haven, Connecticut, United States; Boston University, Boston, Massachusetts, United States

## Abstract

**Background and Objectives:**

Productive engagement (PE) represents a potential pathway toward successful aging (SA). Using time diary data, this study explored how U.S. older adults structure their daily lives in different productive roles, the impacts of these roles on one specific dimension of SA (self-rated health), and the extent to which these patterns differ by race and gender.

**Research Design and Methods:**

We use data from the American Time Use Survey (n = 17,990) and sequence and cluster analyses to identify distinctive daily PE time-use patterns. Multivariable linear regression models were used to evaluate associations between these patterns and self-rated health, and the moderating roles of race and gender.

**Results:**

Five PE clusters were identified: Low Degree of PE (29%), Moderate Unpaid Work & Light Social Participation (42%), Persistent Unpaid Work (12%), Persistent Paid Work (9%), and Persistent Social Participation (7%). White women were the most likely, while Black men were the least likely to have any PE. All PE clusters were positively associated with self-rated health, compared to “Low Degree of PE”. The strongest positive association was observed for “Persistent Paid Work,” especially for women. Racial differences were more prominent among women than men. The health benefits of PE were less pronounced among Black women than White women.

**Discussion and Implications:**

The positive association between PE and self-rated health varies across race-gender groups. Future research should consider the persistent structural barriers that may prevent older adults with disadvantaged backgrounds, particularly Black women, from benefiting from PE and experiencing SA.

